# Supplementary material for: Exploring the Hemostatic Effects of Platelet Lysate-Derived Vesicles: Insights from Mouse Models
Source: Int J Mol Sci. 2024 Jan 18;25(2):1188. doi: 10.3390/ijms25021188 (PMC10816445; doi:10.3390/ijms25021188)
Supplement: Supplementary file 1 [file ijms-25-01188-s001.zip › ijms-2755473-supplementary.pdf]

Supplementary Materials

Table S1. Washed-platelets samples and platelet lysate-derived vesicles

Sample	Unit	w-Plt	20K-vesicles			100K-vesicles		
			Particle concentrations	Mean	Mode	Particle Concentrations	Mean	Mode
		× 10^4/μL	particles/mL	nm	nm	particles/mL	nm	nm
A	3	143	$2.8×10^{12}±2.8×10^{10}$	149±1	142±7	$5.7×10^{11}±4.0×10^{10}$	116±2	97±7
B	3	120	$1.5×10^{12}±3.9×10^{10}$	153±3	136±20	$2.3×10^{11}±1.2×10^{10}$	101±2	72±15
C	3	134	$2.0×10^{12}±2.9×10^{10}$	158±1	144±2	$7.1×10^{11}±1.3×10^{10}$	112±0.5	90±8

Table S2. Thrombin generation assay

	sample	Lag phase [min]	Peak Thrombin [nM]	Peak time [min]	Velocity-Index	AUC
20K-vesicles	A1	7	342.8	9	171.4	3284.4
	A2	7	329.7	9	164.9	3117.4
	B1	6	500.0	7	500.0	3816.5
	B2	7	434.0	8	434.0	3590.0
	C1	6	477.2	7	477.2	3873.0
	C2	6	478.5	7	478.5	3644.3
100K-vesicles	A1	17	0.0	0		0.0
	A2	16	0.0	0		0.0
	B1	10	93.0	14	23.2	1706.6
	B2	13	17.5	20	2.5	212.8
	C1	12	26.5	19	3.8	474.4
	C2	16	30.7	20	7.7	476.9
PLwo-vesicles	A1	16	0.0	0		0
	A2	16	0.0	0		0
	B1	18	0.0	0		0
	B2	19	0.0	0		0
	C1	18	0.0	0		0
	C2	19	0.0	0		0

Lag phase: The time from the addition of reagents to the formation of thrombin.

Peak thrombin: Maximal concentration of thrombin formed.

Peak time: The time from the addition of reagents to the peak thrombin formation.

Velocity-Index: peak thrombin/ (peak time – lag time)

AUC: Area under the curve

We measured each of the three samples twice. Figure 2a was analyzed using sample C1. Despite the small sample sizes, there was no statistically significant difference between 20K-vesicles and 100K-vesicles. However, a clear difference was observed in thrombin formation.

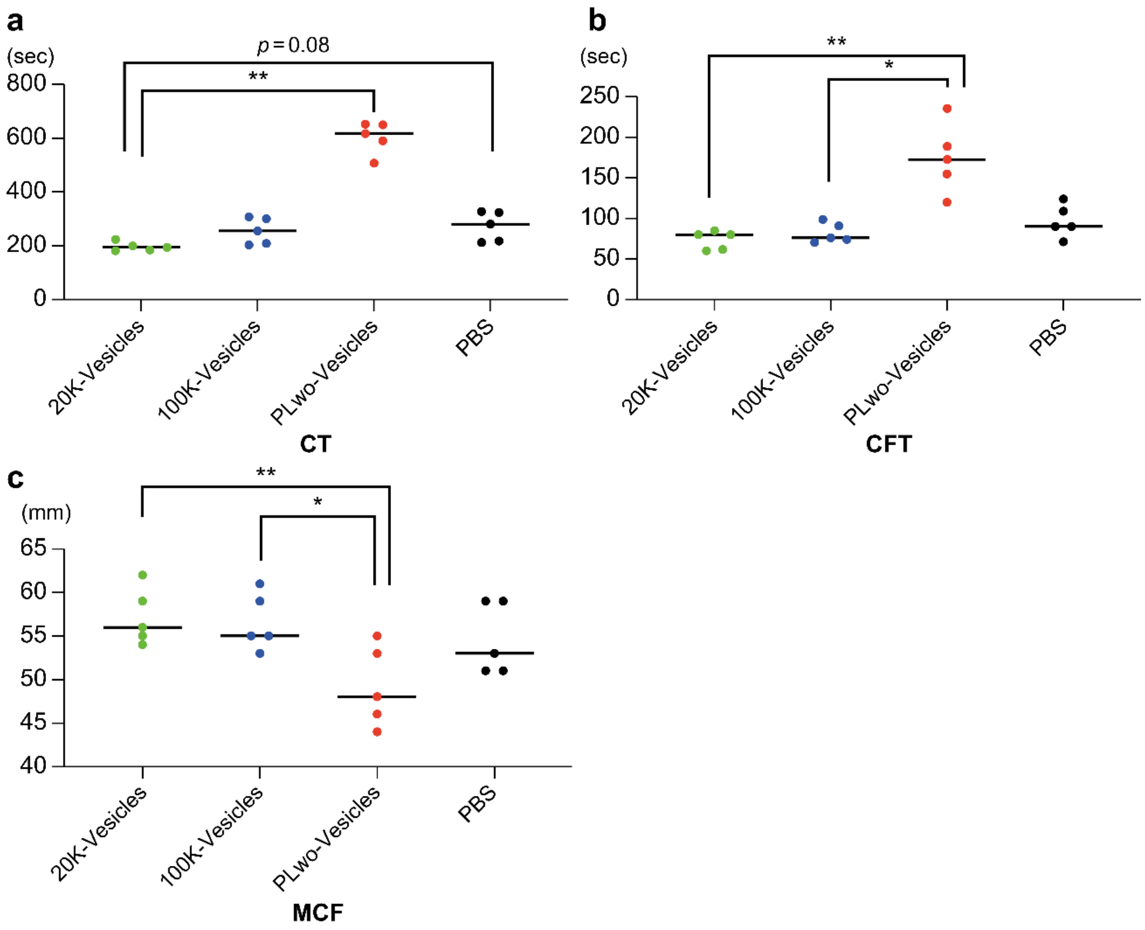

Figure S1. Thromboelastometry parameters in the viscoelastic test. a. CT (Clotting Time), b. CFT (Clot Formation Time), and c. MCF (Maximum Clot Firmness). After the Friedman test, when comparing each parameter using Dunn's multiple comparison test, as illustrated in Supplement 2, significant reductions were observed in the CT and CFT groups between the 20K-vesicles and 100K-vesicles compared to PLwo-vesicles. In the MCF group, there was a significant decrease in PLwo-vesicles compared to 20K-vesicles and 100K-vesicles. * $p < 0.05$, ** $p < 0.01$

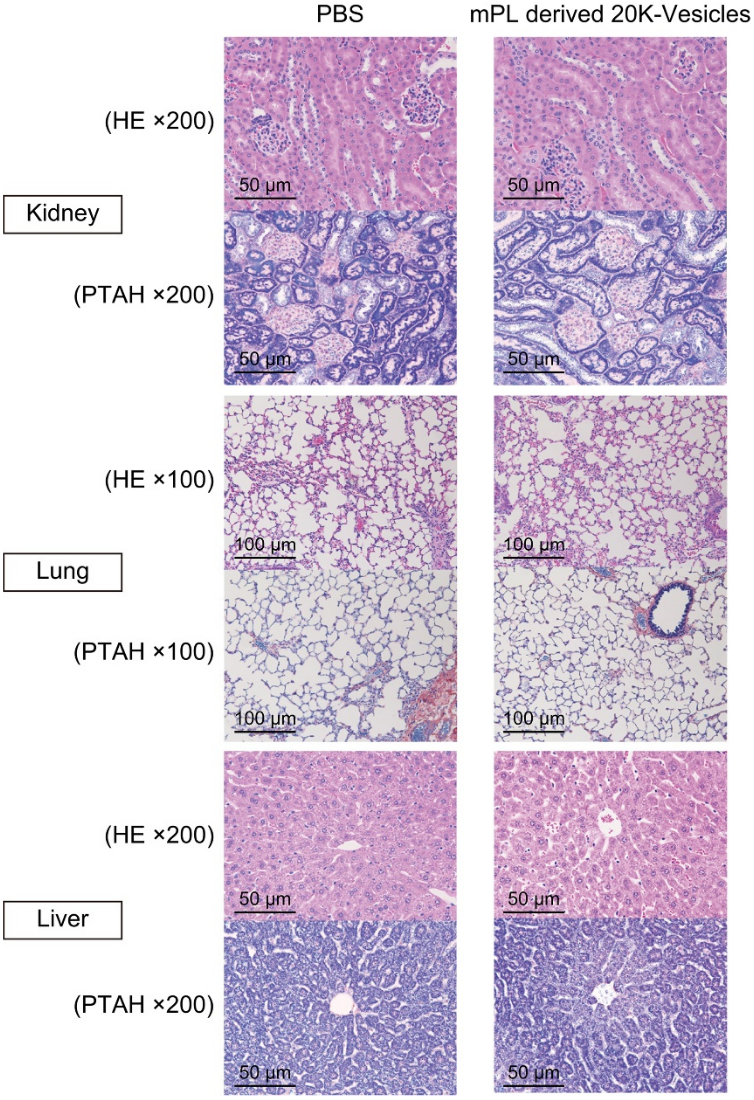

Figure S2. Histological Study. The histopathological findings of the 20K-vesicle administration group were examined 24 hours later by comparing it with the PBS administration group (each group n=5). The specimens were prepared using the maximum sections of the left and right kidneys at the short axis (total of 4 sections), the maximum sections of the two lobes of the liver, and the maximum sections of the left and right lung lobes. HE-stained specimens (hematoxylin and eosin staining) and PTAH-stained specimens (to examine fibrin precipitation) were prepared, and the presence or absence of microthrombi was determined using a blinded method. No thrombus formation was observed in any specimen. Additionally, no other histological findings (changes) were observed in both groups.
